# Transcriptomic analysis reveals the inhibitory effect of beta-sitosterol on proliferation of bovine preadipocytes

**DOI:** 10.1080/10495398.2024.2339406

**Published:** 2024-04-18

**Authors:** Lei Jiang, Yuan Wan, Jinhai Pan, Xiaoyu Mao, Xiaolei Sun, Linsen Zan, Hongbao Wang

**Affiliations:** a College of Animal Science and Technology, Northwest A&F University, Yangling, Shaanxi, China; b National Beef Cattle Improvement Centre, Yangling, Shaanxi, China

**Keywords:** Beta-sitosterol, bovine preadipocyte, RNA-seq, cell cycle, *CCNB1*

## Abstract

Fat deposition affects beef quantity and quality via preadipocyte proliferation. Beta-sitosterol, a natural small molecular compound, has various functions, such as anti-inflammation, antibacterial, and anticancer properties. The mechanism of action of Beta-sitosterol on bovine preadipocytes remains unclear. This study, based on RNA-seq, reveals the impact of Beta -sitosterol on the proliferation of bovine preadipocytes. Compared to the control group, Beta-sitosterol demonstrated a more pronounced inhibitory effect on cell proliferation after 48 hours of treatment than after 24 hours, as evidenced by the results of EdU staining and flow cytometry. RNA-seq and Western Blot analyses further substantiated these findings. Our results suggest that the impact of Beta-sitosterol on the proliferation of bovine preadipocytes is not significant after a 24-hour treatment. It is only after extending the treatment time to 48 hours that Beta-sitosterol may induce cell cycle arrest at the G2/M phase by suppressing the expression of CCNB1, thereby inhibiting the proliferation of bovine preadipocytes.

## Background

The compositional profile, spatial distribution, and lipid composition within the beef exert a direct and discernible impact on both the sensory attributes and nutritional characteristics of the meat.^1^^,^^2^ The proliferation of preadipocytes plays a pivotal role in dictating lipid accretion patterns. Elucidating the complex mechanisms regulating the proliferation of precursor fat cells is crucial, as this may lay an important theoretical foundation for enhancing the production of high-quality beef.^3^

Natural small molecular compounds are organic molecules present in nature, exerting a significant impact on animal growth and development. Many natural small molecule compounds exhibit biological activity, influencing cellular functions, metabolic processes, and the overall physiological state of animals.^4^^,^^5^

Beta-sitosterol, an integral component of the phytosterol ensemble, represents a natural and safe bioactive compound of diminutive molecular dimensions, ubiquitously present in oil-rich botanical specimens.^6^^,^^7^ Research investigations have demonstrated the multifaceted capacities of Beta-sitosterol, encompassing cholesterol-lowering attributes,^8^^,^^9^ blood sugar regulatory effects,^10^^,^^11^ anti-inflammatory properties,^12^^,^^13^ and antioxidative actions.^14^^,^^15^ Furthermore, Beta-sitosterol’s extensive investigation within the realm of colorectal carcinoma, mammary malignancies, and prostatic neoplasms has spotlighted its potential in restraining the canonical Wnt/β-catenin pathway, thereby curbing the proliferation of human colonic malignancies.^16^ Despite the profound focus on Beta-sitosterol’s implications for tumorigenic cell amplification, its role within the realm of adipogenic proliferation, particularly in the context of bovine preadipocytes, remains largely unexplored. Exploring the effect of Beta-sitosterol on the proliferation of bovine preadipocytes may contribute to a deeper understanding of its regulatory role in the biological processes of bovine preadipocytes. Additionally, this research may aid in understanding the development and regulatory mechanisms of adipose tissue, optimizing livestock feeding management, and enhancing meat production efficiency and quality.

The complex network of molecular events directing cellular divisions plays a crucial role in regulating cell cycles. Consisting of different stages including G1, S, G2 and M,^17^ the cell cycle emerges as a crucial regulator governing the path of regular cellular growth and maturation. The Cell cycle is a crucial signaling pathway that plays a pivotal role in regulatory processes, with Cyclin and Cyclin Dependent Kinase (CDK) complexes at its core.^18^ The amalgamation of Cyclin B1 (CCNB1) and Cyclin-dependent kinase 1 (CDK1) forms a decisive juncture in orchestrating the transition from the G2 phase to the culmination of the M phase. Concurrently, CDK1 activity undergoes modulation through a symphony of phosphorylation-based modifications.^19–21^ This research aims to analyze the impact of Beta-sitosterol on the proliferation of bovine preadipocytes based on transcriptome sequencing.

## Methods

### Cell isolation and culture

The cell model selected in this study originates from the preadipocytes of Qinchuan bovine perirenal adipose tissue, known as bovine preadipocytes. Bovine preadipocyte isolation and cell culture were performed as previously described by Wang et al. ^22^ The perirenal adipose tissue of four-day-old male calves was aseptically collected and transported to a sterile cell laboratory. The tissue was washed three times with PBS containing 2% penicillin and streptomycin to remove most of the connective tissue and blood. Subsequently, the tissues were cut into 1–2 mm^3^ pieces and digested with 0.2% type I collagenase for 60 minutes in a water bath shaker at 37 °C. The digested product was neutralized with medium containing 10% FBS and then filtered through 70 and 200 mesh sterile cell sieves. The collected filtrate was centrifuged at 1500 rpm for 10 minutes, and the supernatant was discarded. The cells were resuspended in DMEM/F12 medium (growth medium) containing 10% FBS. Next, the cells were seeded in a culture dish at a density of 2.5 × 10^5^ cells and cultured in an incubator at 37 °C with a concentration of 5% CO_2_.

### Observation of cell morphology

Bovine preadipocytes were seeded in 6-well culture plates, and Beta-sitosterol (dissolved in absolute ethanol. Solubility in ethanol: 5 mg/mL (12.06 mM; sonication-assisted dissolution)) was added at different concentrations (0, 1, 5, 10, 15, 20, 25, 30, 35, 40 μM). The cells were then incubated for 24 and 48 hours. An equal amount of absolute ethanol was added to the control group. Morphological observations were performed using a fluorescent inverted microscope IX71 (Olympus, Japan).

### Cell counting kit-8 assay (CCK-8)

The CCK-8 assay was conducted using CCK-8 (Bioscience, China) following the manufacturer’s instructions. Briefly, after 24 and 48 hours of treatment with Beta-sitosterol, each group had six biological replicates, and 10 μL of CCK-8 solution was added to the normal growth medium to incubate the cells for 2.5 hours. The results were subsequently measured using a microplate reader (Infinite M200 PRO; Tecan, Switzerland).

### 5-Ethynyl-2-deoxyuridine assay (EdU)

To investigate cell proliferation, EdU assays were conducted using the Cell EdU Proliferation Kit (Bioscience, China) following the manufacturer’s instructions. Briefly, after 24 and 48 hours of treatment with Beta-sitosterol, cells were exposed to 2 μM EdU solution in normal growth medium for 3 hours. Subsequently, the cells were fixed with 4% paraformaldehyde and permeabilized with 0.5% Triton X-100. After incubation with EdU working solution for 30 minutes, the cells were treated with Hoechst33342 solution to stain nuclei. Images were acquired using EVOS™ Auto 2 (Thermo Fisher Scientific, USA).

### Cell flow cytometry assay

After 24 and 48 hours of treatment with Beta-sitosterol, staining was conducted using a cell cycle kit (MultiScience, China) following the manufacturer’s instructions. Subsequently, 10,000 cells were analyzed by flow cytometry (BD FACSAria™ III, America) for detection. Data analysis was carried out using ModFit™ LT (version 5.0; Verity Software House, USA).

### RNA-seq

Following 24-hour and 48-hour treatment periods with Beta-sitosterol (Total of 12 samples, with 3 biological replicates per group), the construction of the library was executed utilizing Illumina’s NEBNext® UltraTM RNA Library Prep Kit. Subsequent to sequencing, the raw data underwent filtration using SOAPnuke (v1.5.6)^23^ to eliminate: (1) reads containing adapters (denoting adapter contamination); (2) reads with an unknown base N content exceeding 5%; (3) reads characterized by low quality (where bases with quality values below 15 constitute over 20% of total read bases), yielding refined, clean data. For subsequent analysis, the data was subjected to processing within the Dr. Tom multi-omics data mining system (https://biosys.bgi.com), encompassing analysis, visualization, and mining. Differential gene analysis encompassed aligning the clean data to the reference genome employing HISAT2 (v2.1.0)^24^ software and Bowtie2 (v2.3.4.3)^25^ to align the clean data with the reference gene set provided by the Dr. Tom multi-omics data mining system. Gene expression quantification was achieved through employment of RSEM (v1.3.1)^26^ software. Subsequent generation of a cluster heatmap depicting gene expression variations across distinct samples was realized using pheatmap (v1.0.8).^27^ Differential gene detection was conducted utilizing DESeq2 (v1.4.5)^28^ (under the criterion of Q < 0.05. Further insight into gene functions associated with phenotypic alterations was attained through KEGG and GO enrichment analysis. Leveraging the hypergeometric test, GO (http://www.geneontology.org/) and KEGG (https://www.kegg.jp/) enrichment analyses were performed, setting the threshold at Q < 0.05^29^ for significant enrichment in candidate genes. Moreover, GSEA was executed employing GSEA v3.0.^30^ Additionally, KDA was conducted utilizing Mergeomics 2.0.^31^

### Quantitative reverse transcription-PCR

Total RNA was extracted from bovine preadipocytes using RNAiso Plus (TaKaRa, Japan) following the manufacturer’s instructions. Quantitative reverse transcription PCR (qRT-PCR) was performed using TB Green Premix Ex Taq II (TaKaRa, Japan) on the CFX Connect system (Bio-Rad, USA). The 2^-ΔΔCt^ method was used to calculate the relative expression levels of mRNA, with the β-actin gene serving as an internal control for data normalization. The qRT-PCR primer sequences are listed in Table 1.

**Table 1.** Primer sequences for qRT-PCR verification of RNA-seq results.

Gene/Accession No.	Primer sequences (5′-3′) (Forward/Reverse)	Size(bp)
*HSPA6*/XM_002685850.6	F:GCCTGCCTACTTCAACGACT	169 bp
	R:CCCACCCAGGTCGAAAATGA	
*FOSB*/NM_001102248.1	F:AAGGGAGCTGACTGACCGAC	98 bp
	R:CCTTCTCCTTTTGGAGCTCGG	
*ABCA1*/NM_001024693.1	F:AGCCAGGATCTGTGTCTGGA	124 bp
	R:GTACGCTGATGGCTGGTCA	
*ARRDC3*/NM_001076257.2	F:TCAGCTTCGAGCTTCCACAG	89 bp
	R:GTGCAATTCGGCTTTCACCC	
*CYP3A4*/NM_174531.3	F:AGAGAAAGGCACGTCTGTCG	115 bp
	R:GGGATCTTGTGGGTTGCTGA	
*LSS*/NM_001046564.1	F:CTGGAAGCTCACCTCTTGGG	181 bp
	R:TGATCAGGAGGCCTGGCA	
*SQLE*/NM_001098061.1	F:CGCTGTTTTCCAGGCCAAAA	183 bp
	R:ACCGGACCTGCAACACATTT	
*TNFSF9*/NM_001319902.1	F:CAACTCTCCGCAGCAGGG	114 bp
	R:GCCAGAGTCACACCTGTCAA	
*INSIG1*/NM_001077909.1	F:GTGACGGTGGGGAACATAGG	133 bp
	R:CACTACCCAGTATCGCGGAC	
*SCD*/NM_173959.4	F:ATCCGACCTAAGAGCCGAGA	109 bp
	R:CCACAGATACCATGGCACGA	
*CCL5*/NM_175827.2	F:CACCCACGTCCAGGAGTATT	154 bp
	R:TCCTCCACCCTAGCTCAACT	
*KIT*/NM_001166484.1	F:AATGTGAAGCGCGAGTACCA	130 bp
	R:ACACAGACACAACTGGCACA	
*MEOX1*/NM_001035376.2	F:CTATGAGGTCCTTGGGAGCAC	199 bp
	R:GAGTCGGGTCAGGTAGTTGTG	
*HMGN5*/XM_002699936.6	F:AGCTGCAACAATGCCCAAAAG	103 bp
	R:TATAATGGGCACAGGCAAAGC	
*NRIP3*/NM_001102218.2	F:GGTTTCTTGCCAGTGTGCTG	161 bp
	R:TCAGATGCCGGGGTAGAGAA	
*FADS2*/NM_001083444.1	F:ACCGACAAGTGGCTGGTCAT	186 bp
	R:CAGCTCGCCAATTAACAGGG	
*β-actin*/NM_173979.3	F:ATCGGCAATGAGCGGTTC	144 bp
	R:CGTGTTGGCGTAGAGGTC	

### Western blot analysis

Cells were washed with PBS and then digested with 0.25% trypsin (Gibco, USA). Total cellular protein was extracted using lysis buffer and PMSF (Beyotime, China). Protein electrophoresis was conducted on a 12% SDS-PAGE gel with a constant current of 260 mA. The proteins were then transferred to a PVDF membrane, and QuickBlock™ Western blocking solution was used to block the membrane to prevent nonspecific protein detection. The membrane was incubated with the specific primary antibody overnight at 4 °C, followed by incubation with the secondary antibody at room temperature for 2 hours. Western blot images were obtained using a Bio-Rad molecular imager (Bio-Rad, USA). The images were captured and quantified using the Gel Doc™ XR + system with Image Lab™ software (Bio-Rad). The relevant antibody information is as follows: four primary antibodies (CDK1 [ab275958, 1:2000; Abcam, Cambridge, UK], Phospho-CDK1 (Tyr15) [AF3108, 1:500, Affinity, USA], CCNB1 [55,004-1-AP, 1:1000, Proteintech, China], β-actin [6008-1-Ig, 1:5000, Proteintech, China]), and one secondary antibody (ab150077, 1:5000, Abcam, Cambridge, UK).

### Statistical analysis

All data are presented as means ± SEM (standard error of the mean) of at least three independent experiments and analyzed using the *t*-test in GraphPad Prism 9. Means were considered statistically significant at *p* < 0.05 and 0.01 (*p < 0.05; **p < 0.01).

## Results

### Effect of beta-sitosterol on morphology and viability of bovine preadipocytes

**Figure 1.**
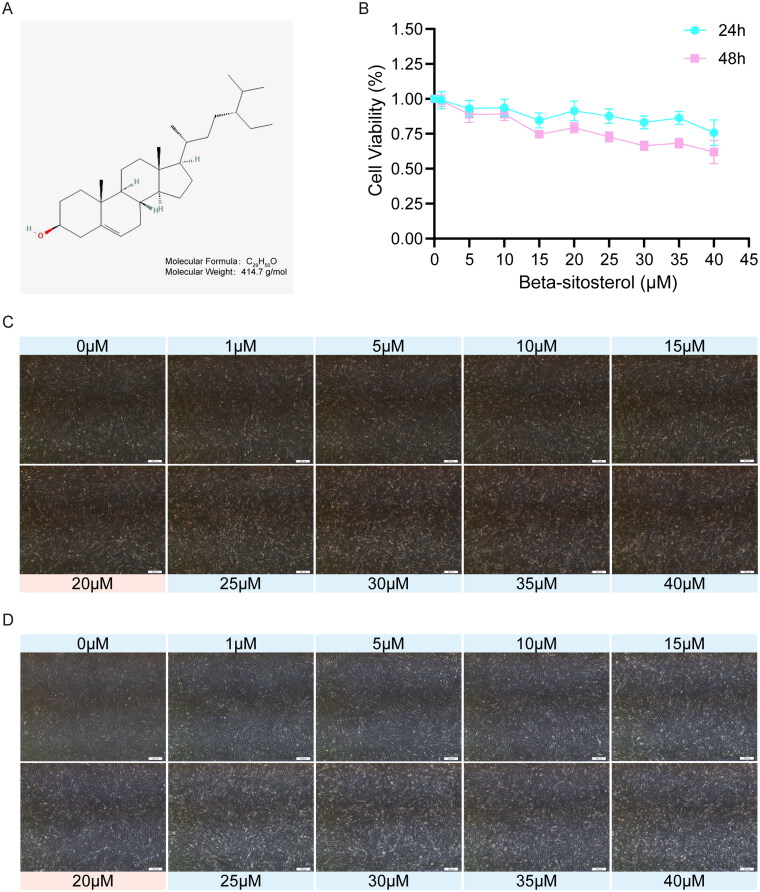
Effect of beta-sitosterol the morphology and viability of bovine preadipocytes. (A) Molecular weight and structural formula of beta-sitosterol. (B) Cell viability curves of bovine preadipocytes treated with beta-sitosterol at different concentrations (0, 1, 5, 10, 15, 20, 25, 30, 35, 40 μM) for 24 and 48 hours. (C) Cell morphology of bovine preadipocytes treated with beta-sitosterol at different concentrations (0, 1, 5, 10, 15, 20, 25, 30, 35, 40 μM) for 24 hours. (D) Cell morphology of bovine preadipocytes treated with beta-sitosterol at different concentrations (0, 1, 5, 10, 15, 20, 25, 30, 35, 40 μM) for 48 hours.

The molecular weight and structure of Beta-sitosterol are shown in Fig. 1A, and the information comes from Pubchem (https://pubchem.ncbi.nlm.nih.gov). To determine the appropriate concentration for subsequent experiments, the cell viability of bovine preadipocytes treated with different concentrations of Beta-sitosterol for 24 and 48 hours was assessed using the CCK-8 assay. Simultaneously, preadipocytes were exposed to various concentration gradients of Beta-sitosterol for 24 and 48 hours, and the cell morphology was observed. The results indicate that concentrations of Beta-sitosterol greater than 20 μM significantly affect cell viability (Fig. 1B) and cell morphology (Fig. 1C,D). As a result, concentrations exceeding 20 μM were excluded from the test, and a concentration of 20 μM was selected for subsequent experiments.

### Beta-sitosterol inhibits the proliferation of bovine preadipocytes

**Figure 2.**
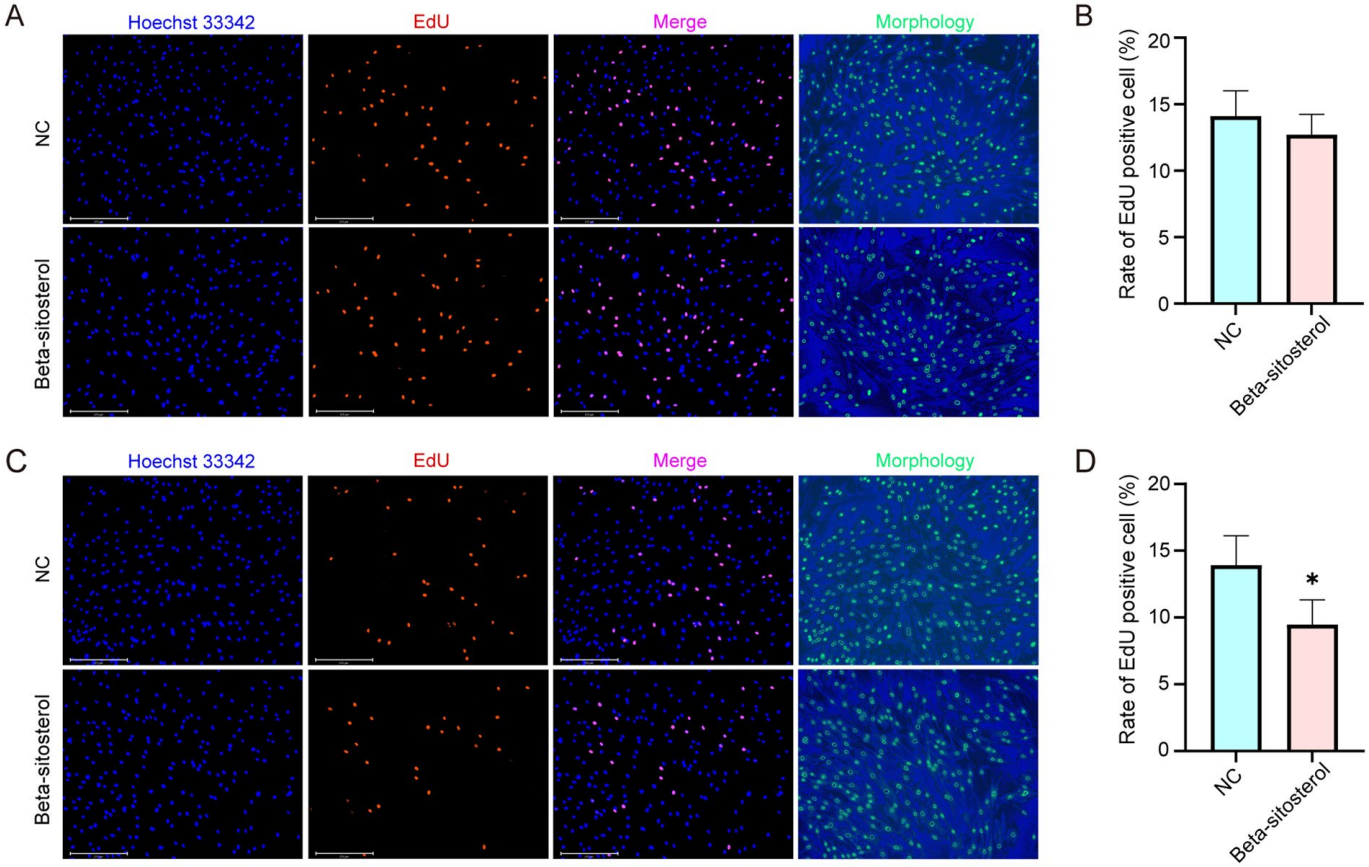
Beta-sitosterol inhibits the proliferation of bovine preadipocytes. (A) EdU staining of bovine preadipocytes treated with 20 μM beta-sitosterol for 24 hours. (B) The quantitative analysis of EdU results after 24 hours of beta-sitosterol treatment. (C) EdU staining of bovine preadipocytes treated with 20 μM beta-sitosterol for 48 hours. (D) The quantitative analysis of EdU results after 48 hours of beta-sitosterol treatment. (*p < 0.05).

After 24-hour and 48-hour treatment with 20 µM beta-sitosterol, we conducted EdU staining. The results revealed that following 24 hours of treatment with 20 µM Beta-sitosterol, there was no significant difference in the proportion of EdU positive cells between the NC group and the Beta-sitosterol group (Fig. 2A,B). After 48 hours of treatment, compared to the NC group, the proportion of EdU-positive cells in the Beta-sitosterol group significantly decreased (*p* < 0.05) (Fig. 2C,D). These findings suggest that 20 µM Beta-sitosterol may have an inhibitory effect on the proliferation of bovine preadipocytes, and there may be a time-dependent aspect to this inhibition.

### Beta-sitosterol inhibits the transition from G2 to M phase of the cell cycle of bovine preadipocytes

**Figure 3.**
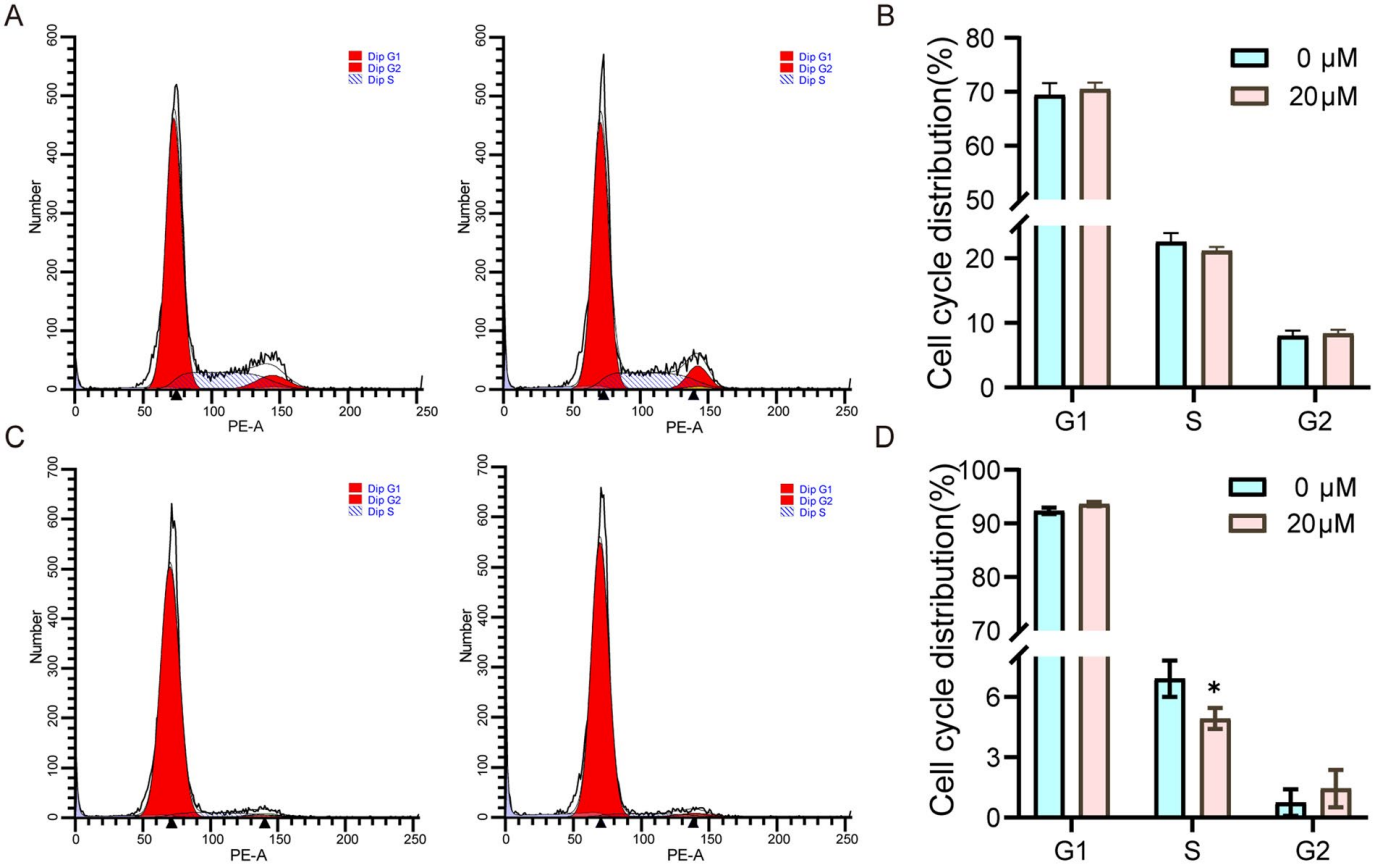
Beta-sitosterol inhibits the transition from G2 to M phase of the cell cycle of bovine preadipocytes. (A) Flow cytometry analysis of bovine preadipocytes after treatment with 20 μM Beta-sitosterol for 24 hours, showing the cell cycle distribution in the NC group (left) and beta-sitosterol-treated group (right). (B) Quantitative analysis of flow cytometry results for bovine preadipocytes after 20 μM Beta-sitosterol treatment for 24 hours. (C) Flow cytometry analysis of bovine preadipocytes after treatment with 20 μM Beta-sitosterol for 48 hours, showing the cell cycle distribution in the NC group (left) and beta-sitosterol-treated group (right). (D) Quantitative analysis of flow cytometry results for bovine preadipocytes after 20 μM Beta-sitosterol treatment for 48 hours. (*p < 0.05).

To further observe the inhibitory effect of Beta-sitosterol on the proliferation of bovine preadipocytes, we investigated the influence of Beta-sitosterol on the cell cycle of bovine preadipocytes. we performed cell cycle analysis using flow cytometry after treating the cells with 20 μM Beta-sitosterol for 24 and 48 hours. Notably, no significant difference was observed in the cell cycle of bovine preadipocytes after Beta-sitosterol treatment for 24 hours (Fig. 3A,B). However, after 48 hours of treatment, compared with the NC group, the proportion of cells in S phase was significantly decreased in the Beta-sitosterol group (*p* < 0.05), and the proportion of cells in G2 phase tended to increase (Fig. 3C,D). The results indicate that treatment with 20 μM Beta-sitosterol for 24 hours does not significantly affect the cell cycle of bovine preadipocytes. However, after 48 hours of treatment, it inhibits the transition of bovine preadipocytes from the G2 phase to the M phase. Interestingly, this observed effect also exhibits time dependency.

### Transcriptome sequencing and analysis

**Figure 4.**
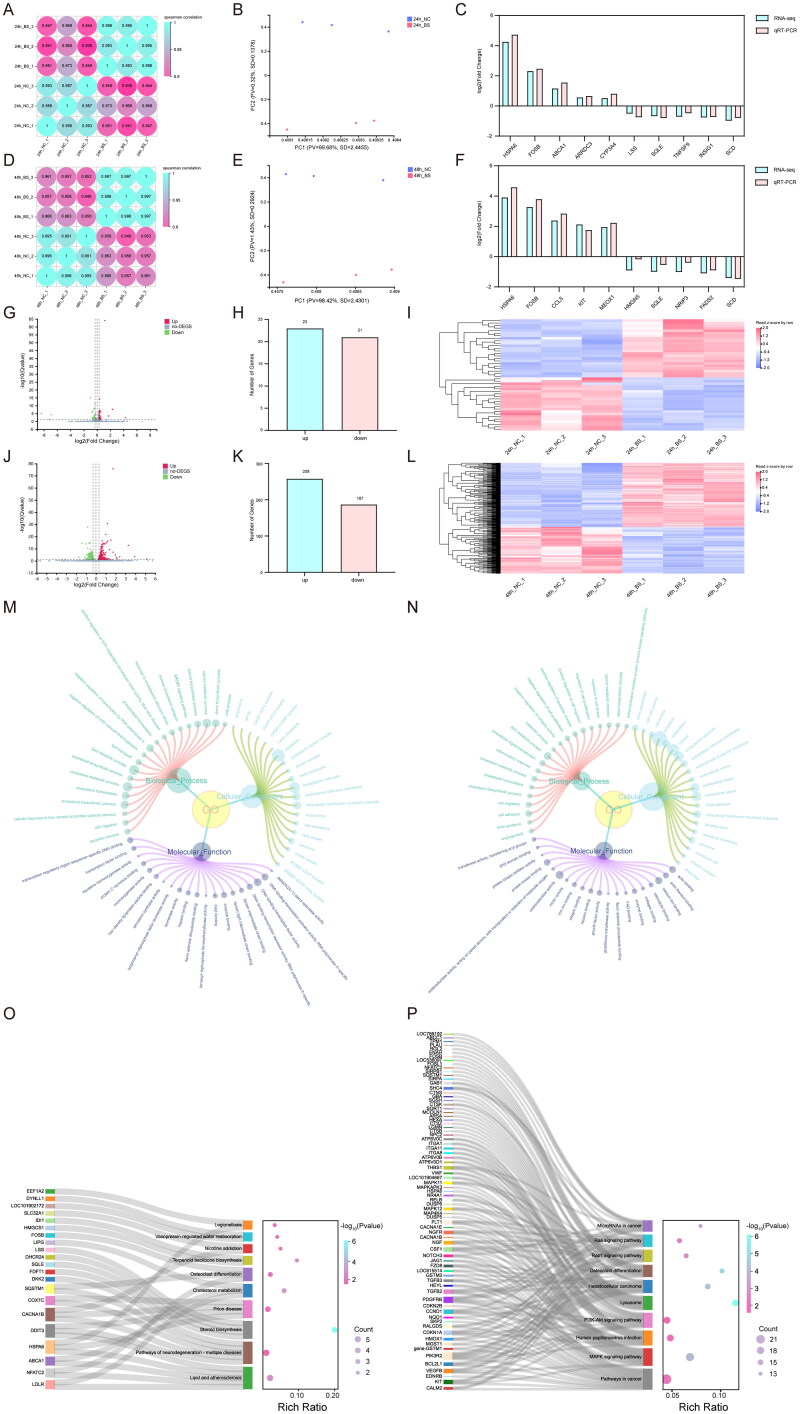
Transcriptome sequencing and analysis. (A) Correlation analysis of RNA-seq data from bovine preadipocytes treated with 20 μM beta-sitosterol for 24 hours. (B) PCA of RNA-seq data from bovine preadipocytes treated with beta-sitosterol for 24 hours. (C) Validation of RNA-seq results by qRT-PCR after beta-sitosterol treatment of bovine preadipocytes for 24 hours. (D) Correlation analysis of RNA-seq data from bovine preadipocytes treated with 20 μM beta-sitosterol for 48 hours. (E) PCA of RNA-seq data from bovine preadipocytes treated with beta-sitosterol for 48 hours. (F) Validation of RNA-seq results by qRT-PCR after beta-sitosterol treatment of bovine preadipocytes for 48 hours. (G) Volcano plot displaying differentially expressed genes (DEGs) after beta-sitosterol treatment of bovine preadipocytes for 24 hours, with red dots indicating up-regulated genes and green dots indicating down-regulated genes. (H) Statistical results of volcano plot after beta-sitosterol treatment of bovine preadipocytes for 24 hours. (I) Clustering heatmap analysis of DEGs after beta-sitosterol treatment of bovine preadipocytes for 24 hours. (J) Volcano plot displaying DEGs after beta-sitosterol treatment of bovine preadipocytes for 48 hours. (K) Statistical results of volcano plot after beta-sitosterol treatment of bovine preadipocytes for 48 hours. (L) Clustering heatmap analysis of DEGs after beta-sitosterol treatment of bovine preadipocytes for 48 hours. (M) GO enrichment analysis results of DEGs in bovine preadipocytes treated with beta-sitosterol for 24 hours. (N) GO enrichment analysis results of DEGs in bovine preadipocytes treated with beta-sitosterol for 48 hours. (O) KEGG enrichment analysis results of DEGs after beta-sitosterol treatment of bovine preadipocytes for 24 hours. (P) KEGG enrichment analysis results of DEGs after beta-sitosterol treatment of bovine preadipocytes for 48 hours.

To further elucidate the potential mechanism underlying the inhibitory effect of Beta-sitosterol on bovine preadipocyte proliferation, we treated the cells with 20 μM Beta-sitosterol for 24 and 48 hours, followed by RNA-seq analysis (Sequence Read Archive, SRA No. SRP457194). Correlation analysis and PCA results demonstrated a significant grouping of the NC group and the Beta-sitosterol treatment group at both time points (Fig. 4A,B,D,E). Comparing the Beta-sitosterol treatment group with the NC group after 24 hours of treatment, we identified 44 differentially expressed genes (DEGs) (Q < 0.05, |log_2_FC| >0.3) (Fig. 4G), of which 23 genes were up-regulated and 21 genes were down-regulated (Fig. 4H,I). This indirectly reflects a minor impact of 24-hour treatment with Beta-sitosterol on bovine preadipocytes. After 48 hours of treatment, we identified 445 DEGs (Q < 0.05, |log_2_FC| >0.3) (Fig. 4J), with 258 genes up-regulated and 187 genes down-regulated (Fig. 4K,L). To validate the RNA-seq data, we selected the top 5 up-regulated and down-regulated genes and confirmed their mRNA expression changes using RT-qPCR. The expression levels of all these genes were consistent with the RNA-seq results (Fig. 4C,F), confirming the reliability of the RNA-seq findings. GO analysis revealed that after 24 hours of treatment, the DEGs were mainly involved in biological processes, such as lipid metabolic process, cholesterol metabolic process, steroid metabolic process, cholesterol biosynthetic process, and sterol biosynthetic process (Fig. 4M). After 48 hours of treatment, the DEGs were mainly associated with biological processes including lipid metabolic process, intracellular signal transduction, cholesterol metabolic process, positive regulation of apoptotic process, and negative regulation of cell proliferation (Fig. 4N). This implies that Beta-sitosterol may impact biological processes related to cell proliferation. Furthermore, KEGG analysis indicated that after 24 hours of treatment, the major enrichment signaling pathways of DEGs were related to Lipid and atherosclerosis, Steroid biosynthesis, and Cholesterol metabolism (Fig. 4O). After 48 hours of treatment, the major enrichment signaling pathways of DEGs included Pathways in cancer, MAPK signaling pathway, PI3K-Akt signaling pathway and Cell cycle (Fig. 4P). These findings suggest that Beta-sitosterol may play a role in the proliferation of bovine preadipocytes through the above-mentioned signaling pathways.

### Beta-sitosterol inhibits the proliferation of bovine preadipocytes through the Cell cycle

**Figure 5.**
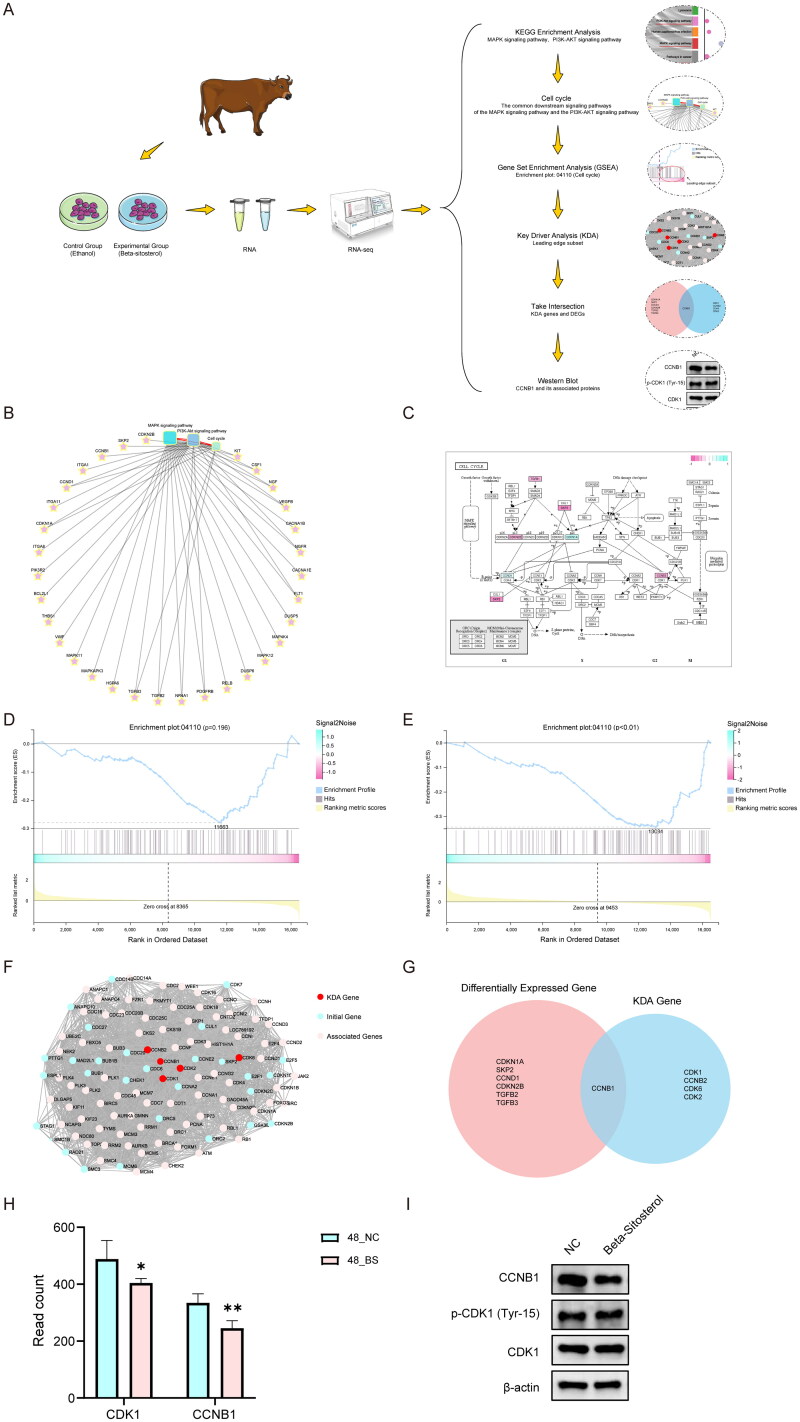
Beta-sitosterol inhibits the proliferation of bovine preadipocytes through the cell cycle. (A) Analysis flow chart. (B) Network diagram depicting the KEGG analysis results of MAPK, PI3K-akt, and cell cycle after treating bovine preadipocytes with 20 μM beta-sitosterol for 48 hours. (C) Enrichment of differentially expressed genes (DEGs) in the cell cycle after 48 hours of 20 μM beta-sitosterol treatment in bovine preadipocytes. (D) GSEA results of cell cycle in RNA-seq after bovine preadipocytes were treated with 20 μM beta-sitosterol for 24 hours. (E) GSEA results of cell cycle in RNA-seq after treating bovine preadipocytes with 20 μM beta-sitosterol for 48 hours. (F) KDA results of leading-edge subsets in cell cycle GSEA after bovine preadipocytes were treated with 20 μM beta-sitosterol for 48 hours. (G) Venn diagrams illustrating the intersection between DEGs associated with the cell cycle and KDA genes after 48 hours of 20 μM beta-sitosterol treatment in bovine preadipocytes. (H) The read count of *CCNB1* and *CDK1* in RNA-seq. (I) Western blot analysis of relative protein expression levels of CDK1, Phospho-CDK1 (Tyr15), and CCNB1 after 48 hours of 20 μM beta-sitosterol treatment in bovine preadipocytes.(*p < 0.05, **p < 0.01).

The analysis flowchart can be found in Fig. 5A. After treating bovine preadipocytes with 20 µM of Beta-sitosterol for 48 hours, KEGG enrichment analysis revealed significant enrichment of DEGs primarily in signaling pathways such as MAPK and PI3K-Akt. Both of these signaling pathways can influence downstream Cell cycle. (Fig. 5B). The enrichment of DEGs in the Cell cycle is depicted in Fig. 5C. To further investigate the impact on the Cell cycle, we conducted GSEA, which indicated that Beta-sitosterol treatment for 24 hours tended to inhibit the Cell cycle (Fig. 5D). However, after 48 hours of treatment, there was a significant inhibition of the Cell cycle (*p* < 0.01) (Fig. 5E). This result suggests that the impact of Beta-sitosterol on the Cell cycle may exhibit time dependency. After treating bovine preadipocytes with 20 μM Beta-sitosterol for 48 hours, we performed Key driver analysis (KDA) on the Leading edge subsets identified in GSEA of the Cell cycle, ultimately identifying 5 key driver genes (KDA genes) (Fig. 5F). The intersection of KDA genes and DEGs revealed *CCNB1* as the sole common gene. (Fig. 5G). Notably, CCNB1 is a cyclin that controls the transition from the G2 phase to the M phase of the cell cycle, which is consistent with the previously mentioned cell flow cytometry assay results. The read count information for *CCNB1* and *CDK1* is depicted as shown in Fig. 5H. To further validate this finding at the protein level, we performed Western blot analysis targeting CCNB1 and its associated proteins (CDK1 and Phospho-CDK1 (Tyr15)). The research results indicate that Beta-sitosterol effectively inhibits the expression of CCNB1, while no substantial changes were observed in the protein expression levels of CDK1 and Phospho-CDK1 (Tyr15) (Fig. 5I). In summary, the results indicate a trend of inhibiting the cell cycle in bovine preadipocytes after 24 hours of treatment with Beta-sitosterol, although it is not significant. After 48 hours of treatment, it appears to inhibit the transition from the G2 phase to the M phase of the cell cycle by suppressing the expression of CCNB1, thereby inhibiting the proliferation of bovine preadipocytes.

## Discussion

The proliferation of preadipocytes constitutes a pivotal determinant in the context of adipose deposition. Natural small molecular compounds exert a discernible impact on this specific stage. For instance, Grape Seed Procyanidin Extract (GSPE) has been observed to inhibit the proliferation of porcine preadipocytes, inducing a halt in the cell cycle progression.^32^ Similarly, Black Adzuki Bean Extract (BABE) and Purple Sweet Potato Leaf Extract (PSPLE) display a capacity to moderately suppress the proliferation of 3T3-L1 cells.^33^^,^^34^ These findings collectively indicate a plausible interconnection between natural small-molecule compounds and the intricate landscape of fat deposition.

Beta-sitosterol, a naturally occurring small molecule compound found in the form of free sterol ester, or stanol ester,in leaves, rhizomes and fruits of various plants,^35^ previous research has highlighted the pivotal role of Beta-sitosterol in cancer cell proliferation, demonstrating its ability to suppress the proliferation of various types of cancer cells.^36–38^ This study reveals the impact of Beta-sitosterol on the proliferation of bovine preadipocytes. Treatment of bovine preadipocytes with 20 μM Beta-sitosterol for 24 hours had no significant effect on the proliferation of bovine preadipocytes. The treatment of bovine preadipocytes with 20 μM Beta-sitosterol for 48 hours significantly inhibits the proliferation of bovine preadipocytes.

In the cell cycle, the transition from the G2 phase to the M phase serves as a pivotal checkpoint, determining whether cells proceed into the mitotic phase. This transition process is regulated by multiple signaling pathways, involving a series of crucial protein kinases and cell cycle proteins. The G2/M transition is regulated by cyclin-dependent kinases (CDKs), a class of protein kinases whose activity depends on specific cyclin binding. At the end of the G2 phase, CDK1 binds to its cyclin partner, cyclin B, reaching peak levels, forming the active complex CDK1-cyclin B. This complex regulates key steps in cell entry into mitosis.^39^^,^^40^ Additionally, the G2/M transition is regulated by various phosphatases. As a natural compound with multiple targets, Beta-sitosterol modulates the activity of various cyclin–CDK complexes to induce cell cycle arrest.^41^ In human oral cancer research, Beta-sitosterol inhibits the expression of CCNB1.^42^ In this study, we conducted transcriptome sequencing analysis to identify key driver genes involved in Beta-sitosterol-mediated inhibition of bovine preadipocyte proliferation. The analysis revealed a significant reduction in the expression level of CCNB1 following Beta-sitosterol treatment. However, there were no significant changes observed in the expression level of CDK1 or the phosphorylation level of CDK1(Tyr-15). This may limit the ability to form the active complex CDK1-cyclin B. In summary, Beta-sitosterol likely inhibits the transition of the cell cycle from the G2 phase to the M phase by downregulating the expression level of CCNB1, ultimately suppressing the proliferation of bovine preadipocytes.

Until now, there have been relatively few reports on the effects of Beta-sitosterol on preadipocyte proliferation in cattle. Our experimental results suggest that after 24 hours of Beta-sitosterol treatment, there is a trend of inhibiting cell proliferation; however, a significant inhibitory effect on cell proliferation is observed only after 48 hours of treatment. This indicates that the impact of Beta-sitosterol on bovine preadipocytes may exhibit time dependency. Sometimes, biological responses may require a certain amount of time to manifest and may not be easily observed at earlier time points. This time-dependent effect could be related to factors such as the mechanism of action of small molecules, the cell cycle, and cell type.^43^

Our research suggests that Beta-sitosterol may exert an inhibitory effect on the proliferation of preadipocytes by suppressing the expression of CCNB1, and this effect may be time-dependent. This may establish a certain theoretical foundation for the production of high-quality beef. Although the experimental results presented validate our proposed hypothesis, there are certain aspects that necessitate further investigation. Additionally, the interaction between Beta-sitosterol and key regulatory molecules within the Cell cycle remains to be elucidated. In the future, our research efforts should be directed toward addressing the aforementioned gaps.

## Conclusion

The findings suggest that the impact of Beta-sitosterol treatment on the proliferation of bovine preadipocytes is not substantial within the initial 24-hour period. When the treatment duration is extended to 48 hours, this becomes apparent. During this period, it is observed that Beta-sitosterol induces cell cycle arrest at the G2/M phase by suppressing the expression of CCNB1, thereby further inhibiting the proliferation of bovine preadipocytes. The effectiveness of Beta-sitosterol appears to require a certain duration for accumulation or initiation of biological effects.

## Data Availability

The transcriptome sequencing data of the 12 samples in this experiment have been deposited in the Sequence Read Archive (SRA) database (BioProject: PRJNA1009509(https://dataview.ncbi.nlm.nih.gov/object/PRJNA1009509?reviewer=50a1p5bc1356t2th76hvs1qif2))

## References

[1] Du M, Huang Y, Das A, et al. Manipulating mesenchymal progenitor cell differentiation to optimize performance and carcass value of beef cattle. *J Anim Sci*. 2013;91(3):1–13. 23100595 10.2527/jas.2012-5670

[2] Hausman G, Basu U, Wei S, Hausman D, Dodson M. Preadipocyte and adipose tissue differentiation in meat animals: influence of species and anatomical location. *Annu Rev Anim Biosci*. 2014;2(1):323–351. 25384146 10.1146/annurev-animal-022513-114211

[3] Schumacher M, DelCurto-Wyffels H, Thomson J, Boles J. Fat deposition and fat effects on meat quality—a review. *Animals.* 2022;12(12):1550. 35739885 10.3390/ani12121550 PMC9219498

[4] Guo Y, Ding S-J, Ding X, et al. Effects of selected flavonoids on cell proliferation and differentiation of porcine muscle stem cells for cultured meat production. *Food Res Int*. 2022;160:111459. 36076368 10.1016/j.foodres.2022.111459

[5] Xia G, Sun J, Fan Y, et al. β-sitosterol attenuates high grain diet-induced inflammatory stress and modifies rumen fermentation and microbiota in sheep. *Animals.* 2020;10(1):171. 31963945 10.3390/ani10010171 PMC7022687

[6] Feng S, Belwal T, Li L, Limwachiranon J, Liu X, Luo Z. Phytosterols and their derivatives: Potential health-promoting uses against lipid metabolism and associated diseases, mechanism, and safety issues. *Compr Rev Food Sci Food Saf*. 2020;19(4):1243–1267. 33337101 10.1111/1541-4337.12560

[7] Babu S, Jayaraman S. An update on β-sitosterol: A potential herbal nutraceutical for diabetic management. *Biomed Pharmacother*. 2020;131:110702. 32882583 10.1016/j.biopha.2020.110702

[8] Yuan L, Zhang F, Jia S, Xie J, Shen M. Differences between phytosterols with different structures in regulating cholesterol synthesis, transport and metabolism in Caco-2 cells. *J Funct Foods*. 2020;65:103715.

[9] Wang X, Huang W, Lei L, et al. Blockage of hydroxyl group partially abolishes the cholesterol-lowering activity of β-sitosterol. *J Funct Foods*. 2015;12:199–207.

[10] Kumar S, Kumar V, Prakash O. Enzymes inhibition and antidiabetic effect of isolated constituents from *Dillenia indica*. *Biomed Res Int*. 2013;2013:382063–382067. 24307994 10.1155/2013/382063 PMC3838843

[11] Babu S, Krishnan M, Rajagopal P, et al. Beta-sitosterol attenuates insulin resistance in adipose tissue via IRS-1/Akt mediated insulin signaling in high fat diet and sucrose induced type-2 diabetic rats. *Eur J Pharmacol*. 2020;873:173004. 32045603 10.1016/j.ejphar.2020.173004

[12] Ding K, Tan YY, Ding Y, et al. β-Sitosterol improves experimental colitis in mice with a target against pathogenic bacteria. *J Cell Biochem*. 2019;120(4):5687–5694. 30548286 10.1002/jcb.27853

[13] Sun Y, Gao L, Hou W, Wu J. β-Sitosterol alleviates inflammatory response via inhibiting the activation of ERK/p38 and NF-κB pathways in LPS-exposed BV2 cells. *Biomed Res Int*. 2020;2020:7532306. 32596368 10.1155/2020/7532306 PMC7273476

[14] Devaraj E, Roy A, Royapuram Veeraragavan G, et al. β-Sitosterol attenuates carbon tetrachloride–induced oxidative stress and chronic liver injury in rats. *Naunyn Schmiedebergs Arch Pharmacol*. 2020;393(6):1067–1075. 31930431 10.1007/s00210-020-01810-8

[15] Ortiz-Escarza JM, Medina ME, Trigos A. On the peroxyl radical scavenging ability of β-sitosterol in lipid media: a theoretical study. *J Phys Org Chem*. 2021;34(1):e4123.

[16] Gu S, Liu F, Xie X, et al. β-Sitosterol blocks the LEF-1-mediated Wnt/β-catenin pathway to inhibit proliferation of human colon cancer cells. *Cell Signal*. 2023;104:110585. 36603684 10.1016/j.cellsig.2022.110585

[17] Nasmyth K. Putting the cell cycle in order. *Science*. 1996;274(5293):1643–1645. 8984634 10.1126/science.274.5293.1643

[18] Morgan DO. Cyclin-dependent kinases: engines, clocks, and microprocessors. *Annu Rev Cell Dev Biol*. 1997;13(1):261–291. 9442875 10.1146/annurev.cellbio.13.1.261

[19] Nakanishi M, Ando H, Watanabe N, et al. Identification and characterization of human Wee1B, a new member of the Wee1 family of Cdk-inhibitory kinases. *Genes Cells*. 2000;5(10):839–847. 11029659 10.1046/j.1365-2443.2000.00367.x

[20] Mueller PR, Coleman TR, Kumagai A, Dunphy WG. Myt1: a membrane-associated inhibitory kinase that phosphorylates Cdc2 on both threonine-14 and tyrosine-15. *Science*. 1995;270(5233):86–90. 7569953 10.1126/science.270.5233.86

[21] McGowan CH, Russell P. Human Wee1 kinase inhibits cell division by phosphorylating p34cdc2 exclusively on Tyr15. *Embo J*. 1993;12(1):75–85. 8428596 10.1002/j.1460-2075.1993.tb05633.x PMC413177

[22] Wang Y, Zhang Y, Su X, Wang H, Yang W, Zan L. Cooperative and independent functions of the mir-23a∼ 27a∼ 24-2 cluster in bovine adipocyte adipogenesis. *Int J Mol Sci*. 2018;19(12):3957. 30544847 10.3390/ijms19123957 PMC6321175

[23] Li R, Li Y, Kristiansen K, Wang J. SOAP: short oligonucleotide alignment program. *Bioinformatics*. 2008;24(5):713–714. 18227114 10.1093/bioinformatics/btn025

[24] Kim D, Langmead B, Salzberg SL. HISAT: a fast spliced aligner with low memory requirements. *Nat Methods*. 2015;12(4):357–360. 25751142 10.1038/nmeth.3317 PMC4655817

[25] Langmead B, Salzberg SL. Fast gapped-read alignment with Bowtie 2. *Nat Methods*. 2012;9(4):357–359. 22388286 10.1038/nmeth.1923 PMC3322381

[26] Li B, Dewey CN. RSEM: accurate transcript quantification from RNA-Seq data with or without a reference genome. *BMC Bioinformatics*. 2011;12(1):323. 21816040 10.1186/1471-2105-12-323 PMC3163565

[27] Kolde R, Kolde MR. Package ‘pheatmap. *R Package.* 2015;1(7):790.

[28] Love MI, Huber W, Anders S. Moderated estimation of fold change and dispersion for RNA-seq data with DESeq2. *Genome Biol*. 2014;15(12):550. 25516281 10.1186/s13059-014-0550-8 PMC4302049

[29] Dabney A, Storey JD, Warnes G. Q-value estimation for false discovery rate control. *Medicine.* 2004;344(539):48.

[30] Subramanian A, Tamayo P, Mootha VK, et al. Gene set enrichment analysis: a knowledge-based approach for interpreting genome-wide expression profiles. *Proc Natl Acad Sci USA*. 2005;102(43):15545–15550. 16199517 10.1073/pnas.0506580102 PMC1239896

[31] Ding J, Blencowe M, Nghiem T, et al. Mergeomics 2.0: a web server for multi-omics data integration to elucidate disease networks and predict therapeutics. *Nucleic Acids Res*. 2021;49(W1):W375–W387. 34048577 10.1093/nar/gkab405 PMC8262738

[32] Wei S, Zheng Y, Zhang M, Zheng H, Yan P. Grape seed procyanidin extract inhibits adipogenesis and stimulates lipolysis of porcine adipocytes *in vitro*. *J Anim Sci*. 2018;96(7):2753–2762. 29701782 10.1093/jas/sky158 PMC6095360

[33] Kim M, Park J-E, Song S-B, Cha Y-S. Effects of black adzuki bean (*Vigna angularis*) extract on proliferation and differentiation of 3T3-L1 preadipocytesinto mature adipocytes. *Nutrients.* 2015;7(1):277–292. 25569623 10.3390/nu7010277 PMC4303839

[34] Lee S-L, Lee H-K, Chin T-Y, et al. Inhibitory effects of purple sweet potato leaf extract on the proliferation and lipogenesis of the 3T3-L1 preadipocytes. *Am J Chin Med*. 2015;43(5):915–925. 26205968 10.1142/S0192415X15500536

[35] Gupta E. β-Sitosterol: Predominant phytosterol of therapeutic potential. *Innovat Food Technol Curr Perspect Fut Goals*. 2020;:465–477.

[36] Vundru SS, Kale RK, Singh RP. β-Sitosterol induces G1 arrest and causes depolarization of mitochondrial membrane potential in breast carcinoma MDA-MB-231 cells. *BMC Complement Altern Med*. 2013;13(1):280. 24160369 10.1186/1472-6882-13-280 PMC3819702

[37] Awad AB, Williams H, Fink CS. Phytosterols reduce in vitro metastatic ability of MDA-MB-231 human breast cancer cells. *Nutr Cancer*. 2001;40(2):157–164. 11962251 10.1207/S15327914NC402_12

[38] Sharmila R, Sindhu G. Modulation of angiogenesis, proliferative response and apoptosis by β-sitosterol in rat model of renal carcinogenesis. *Indian J Clin Biochem*. 2017;32(2):142–152. 28428688 10.1007/s12291-016-0583-8 PMC5382068

[39] Clute P, Pines J. Temporal and spatial control of cyclin B1 destruction in metaphase. *Nat Cell Biol*. 1999;1(2):82–87. 10559878 10.1038/10049

[40] Pines J, Hunter T. Human cyclins A and B1 are differentially located in the cell and undergo cell cycle-dependent nuclear transport. *J Cell Biol*. 1991;115(1):1–17. 1717476 10.1083/jcb.115.1.1 PMC2289910

[41] Wang H, Wang Z, Zhang Z, et al. Beta-sitosterol as a promising anticancer agent for chemoprevention and chemotherapy: Mechanisms of action and future prospects. *Adv Nutr*. 2023;14(5):1085–1110. 37247842 10.1016/j.advnut.2023.05.013 PMC10509430

[42] Han CH, Ding H, Casto B, et al. Inhibition of the growth of premalignant and malignant human oral cell lines by extracts and components of black raspberries. *Nutr Cancer*. 2005;51(2):207–217. 15860443 10.1207/s15327914nc5102_11

[43] Ramlugon S, Levendal RA, Frost C. Time-dependent effect of phytocannabinoid treatments in fat cells. *Phytother Res*. 2018;32(6):1080–1089. 29464872 10.1002/ptr.6047

